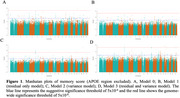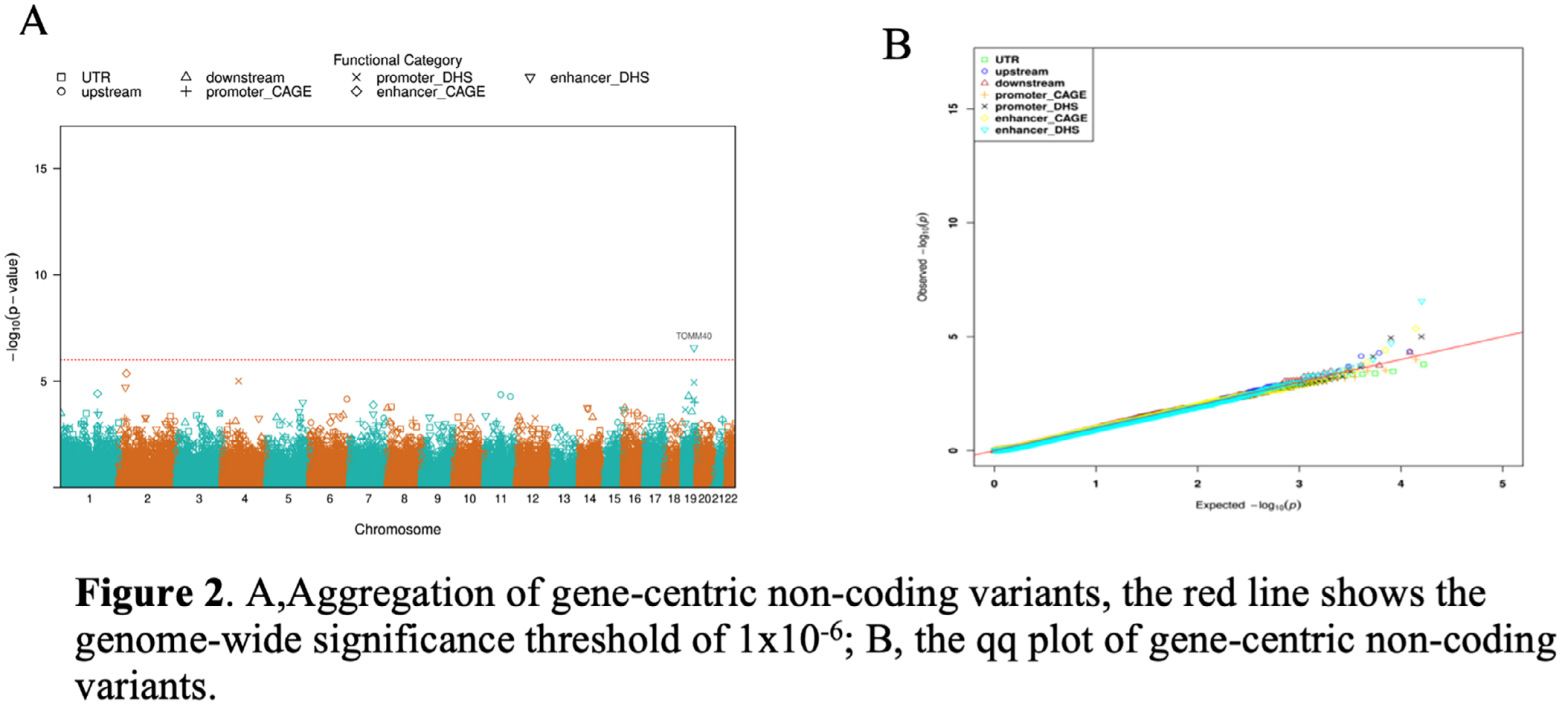# Addressing Heterogeneity in Genetic Studies of Cognitive Function: Insights from the ADSP‐PHC

**DOI:** 10.1002/alz70855_100205

**Published:** 2025-12-23

**Authors:** Jiemin Yang, Dongyu Wang, Andy Rampersaud, Nancy Heard‐Costa, Gina M. Peloso, Seung Hoan Choi, Anita L. DeStefano, Alzheimer Disease Sequencing Project

**Affiliations:** ^1^ Department of Biostatistics, Boston University School of Public Health, Boston, MA, USA; ^2^ Boston University, Boston, MA, USA; ^3^ Research Computing Services, Boston University, Boston, MA, USA; ^4^ Department of Neurology, School of Medicine, Boston University, Boston, MA, USA; ^5^ NHLBI Framingham Heart Study, Framingham, MA, USA

## Abstract

**Background:**

The Alzheimer's Disease Sequencing Project Phenotype Harmonization Consortium (ADSP‐PHC) released harmonized longitudinal cognition data across 12 studies enabling genomic analyses of AD related endophenotypes (ng00067.v11 via NIAGADS). We conducted genome‐wide analyses of common and rare variants using whole genome sequencing (WGS) to identify genetic factors influencing cognitive function.

**Method:**

We selected genetically unique ADSP participants with available cognitive function measures and WGS. After excluding individuals with unknown status or dementia at baseline, 9,698 participants with either mild cognitive impairment or normal cognition remained for a cross‐sectional study of baseline memory score. The analysis began by testing the assocations with common variant (minor allele frequency [MAF] > 0.5%) using linear mixed‐effects model (Model_0) and associated aggregated rare variants (MAF ≤ 0.5%) in both coding and non‐coding regions using STAARpipeline approach. All tests were adjusted for sex, age, sequencing center, study, education, and PCs, with or without APOE status. To address heterogeneity across studies, three models were applied. Model_1 used residuals from memory scores regressed on covariates as the outcome in a linear mixed‐effects model, enabling study‐specific fixed effects. Model_2 allowed for study‐specific residual variance components. Model_3 combined the approaches of Models 1 and 2.

**Result:**

Model_0 yielded a genomic inflation factor (λ) of 1.12. Adjusting for heterogeneity across studies reduced the λ values in all subsequent models: Model_1 (λ=1.11), Model_2 (λ=1.02), Model_3 (λ=1.01) (Figure 1). Across all models, APOE status was associated with baseline memory score. Additionally, a region on chromosome 5 (rs36110370, MAF=0.182), near the *GOLPH3* gene, showed consistent association with lower memory score: Model_0 (β=‐0.054, *p* = 8.6x10^‐8^), Model_1( β=‐0.052, *p* = 1.8x10^‐7^), Model_2 (β=‐0.05, *p* = 1.4x10^‐8^), and Model_3 (β=‐0.047, *p* = 2.6x10^‐8^). Rare noncoding variant analysis revealed an association between *TOMM40* and baseline memory score (Figure 2).

**Conclusion:**

The PHC harmonized memory score retains residual structure which is effectively addressed by modeling study‐specific variance. There was a strong and consistent association of rs36110370 across models, however, the association did not always meet a genome‐wide significance threshold. Analyses of ADSP PHC language and executive scores are ongoing.